# Divergent intestinal barrier and neuroimmune responses to supervised exercise across fibromyalgia, irritable bowel syndrome, and their comorbidity phenotypes: a prospective interventional study

**DOI:** 10.3389/fphys.2026.1912788

**Published:** 2026-08-21

**Authors:** Antonella Bianco, Francesco Russo, Laura Prospero, Gaetana Laselva, Marco Grasso, Benedetta D’Attoma, Nicola Verrelli, Antonia Ignazzi, Isabella Franco, Elisa Grazioli, Attilio Parisi, Giuseppe Riezzo

**Affiliations:** 1 Laboratory of Movement and Wellness, National Institute of Gastroenterology IRCCS “Saverio de Bellis”, Castellana Grotte, Italy; 2 Functional Gastrointestinal Disorders Research Group, National Institute of Gastroenterology IRCCS “Saverio de Bellis”, Castellana Grotte, Italy; 3 Rheumatology Outpatient Clinic, National Institute of Gastroenterology IRCCS “Saverio de Bellis”, Castellana Grotte, Italy; 4 Outpatient Pain Management Unit, National Institute of Gastroenterology IRCCS “Saverio de Bellis”, Castellana Grotte, Italy; 5 University of Rome “Foro Italico”, Rome, Italy

**Keywords:** exercise, fibromyalgia, gut-brain axis, intestinal permeability, irritable bowel syndrome, lactulose/mannitol ratio, phenotype-stratified intervention, zonulin

## Abstract

**Background:**

Fibromyalgia (FM) and irritable bowel syndrome (IBS) are overlapping functional somatic syndromes characterized by gut-brain axis dysregulation and low-grade systemic inflammation. We investigated whether a 16-week supervised combined exercise program is associated with phenotype-specific biomarker changes in patients with FM, IBS, or their comorbidity (FM+IBS).

**Methods:**

In this prospective, single-center, pre-post interventional study, 55 patients (FM, n=15; FM+IBS, n=21; IBS, n=19) completed a 16-week supervised program of aerobic, resistance, and flexibility training three times weekly. Pre- and post-intervention assessments included a panel of intestinal barrier, neuroimmune, and inflammatory biomarkers, together with the Global Physical Capacity Score (GPCS).

**Results:**

In the FM+IBS group, exercise was associated with significant reductions in urinary lactulose excretion (mean delta: -0.160%, p=0.001), L/M ratio (mean delta: -0.011, p=0.026), serum zonulin (mean delta: -6.2 ng/mL, p=0.016), fecal zonulin (mean delta: -65.9 ng/mL, p=0.007), and urinary indoxyl sulfate (mean delta: -23.6 mg/L, p=0.046), with post-intervention L/M values falling below the 0.030 pathological threshold. In the IBS group, serum 5-HT increased (mean delta: +30.0 ng/mL, p=0.028) and IBS-SSS decreased by 85.8 points (p<0.001), exceeding the minimally important difference; both findings were confirmed in a sensitivity analysis restricted to 14 female patients (5-HT: p=0.033; IBS-SSS: p=0.004). In the FM group, IL-6 increased (mean delta: +0.37 pg/mL, p=0.013) and GPCS improved (p=0.047). BDNF, I-FABP, DAO, and LPS remained unchanged across all groups.

**Conclusions:**

Sixteen weeks of combined exercise were associated with phenotype-specific biological patterns. FM+IBS patients showed reductions in barrier-related markers, supported by two independent assays (L/M ratio and indoxyl sulfate), while IBS patients exhibited enhanced serotonergic signaling and symptom relief, both robust to sex-stratified sensitivity analysis. The isolated IL-6 increase in FM patients requires caution, since an acute myokine effect and a chronic inflammatory drift are both compatible with a single measurement taken 16 weeks apart. These exploratory findings, constrained by this proof-of-concept design, support phenotype-stratified randomized controlled trials with active comparators to validate and characterize these differential responses.

## 1 Introduction

Fibromyalgia (FM) and irritable bowel syndrome (IBS) are among the most prevalent functional somatic syndromes, with estimated population prevalence rates of 2-4% and 10-15%, respectively (Wolfe et al., 2010; Mearin et al., 2016). Despite distinct diagnostic criteria, both conditions share key pathophysiological features, including central sensitization, dysregulation of the gut-brain axis, and low-grade systemic inflammation. They co-occur in 30-70% of patients in tertiary care settings (Whitehead et al., 2002). When FM and IBS coexist, symptom burden is amplified, health-related quality of life is substantially impaired, and conventional pharmacological treatments show a less robust response than in either condition alone (Almansa et al., 2009).

The intestinal barrier is a convergence point for the pathophysiological mechanisms underlying both disorders. When tight junction (TJ) integrity is compromised, paracellular translocation of luminal microbial products amplifies visceral and somatic pain via TLR4-mediated neuroinflammatory signaling (Guo et al., 2013; Bischoff et al., 2014). Established markers of barrier dysfunction include the urinary lactulose/mannitol (L/M) ratio and zonulin-related proteins as indices of paracellular permeability (Piche et al., 2009; Fasano, 2011), intestinal fatty acid-binding protein (I-FABP) as a marker of enterocyte injury (Derikx et al., 2008; Adriaanse et al., 2013), and urinary indoxyl sulfate (indican) as a functional readout of luminal protein fermentation and microbial metabolic activity (Farowski et al., 2019).

Throughout this manuscript, we use “intestinal barrier dysfunction” as the umbrella term for the loss of epithelial and tight junction integrity. Within this umbrella, “paracellular permeability” refers specifically to the construct captured by the L/M ratio. The two probes in this test follow different absorption routes: lactulose, a disaccharide, crosses mainly through damaged tight junctions, while mannitol, a smaller monosaccharide, is absorbed mainly through the transcellular and aqueous-pore pathway and serves here to correct for non-mucosal confounders such as gastric emptying and intestinal transit time, not as an independent permeability signal in its own right. Reporting the ratio rather than lactulose excretion alone is the standard way this test isolates the paracellular component from these confounders (Menzies et al., 1999; Piche et al., 2009; Fasano, 2011). We avoid the broader term “intestinal permeability” except where an assay-specific threshold definition requires it, to keep barrier-level and route-specific claims distinct.

In a prior cross-sectional analysis of the same clinical population, we demonstrated that FM+IBS patients exhibit significantly greater barrier dysfunction at baseline than those with either condition alone (Russo et al., 2025). That analysis established the biological starting point for the present intervention study.

Two neuromodulatory systems likely shape the clinical overlap between FM and IBS and mediate exercise effects on these conditions. Serotonin (5-HT), synthesized by enterochromaffin cells, regulates intestinal motility, secretion, and the perception of visceral pain. Downregulation of the serotonin reuptake transporter (SERT) has been documented in both conditions (Mawe and Hoffman, 2013). Brain-derived neurotrophic factor (BDNF) modulates neuronal plasticity and pain sensitization via TrkB signaling in the enteric and central nervous systems and interacts bidirectionally with serotonergic signaling and intestinal barrier integrity (Szuhany et al., 2015).

Among non-pharmacological interventions, physical exercise has substantial evidence supporting its effectiveness for both FM and IBS (Busch et al., 2008; Daley et al., 2008; Häuser et al., 2010). In FM, aerobic and resistance training reduce pain catastrophizing and attenuate HPA axis hyperreactivity. In IBS, moderate-intensity exercise accelerates colonic transit and favorably modulates microbiota composition (Mailing et al., 2019). Proposed mechanisms include exercise-induced attenuation of cortisol-driven barrier disruption and favorable remodeling of tight junction proteins, supported by preclinical and early human data (Dokladny et al., 2016; Feng et al., 2021), and the acute release of IL-6 as an exercise-induced myokine with pleiotropic anti-inflammatory properties (Pedersen and Febbraio, 2008). Whether these biological changes are uniform across phenotypes, or instead depend on the underlying clinical presentation, remains unknown.

Building on our prior cross-sectional data (Russo et al., 2025), we assessed a prespecified biomarker panel covering paracellular permeability, enterocyte injury, microbial metabolic activity, serotonergic signaling, neurotrophic activity, and systemic inflammation before and after 16 weeks of supervised combined exercise training in three diagnostic groups (FM only, IBS only, and FM+IBS comorbidity). We hypothesized that pre-to-post changes in these biomarkers would follow a phenotype-dependent trajectory, reflecting the distinct neuroimmune profiles that characterize each clinical group.

## 2 Methods

### 2.1 Study design and setting

**Figure 1:**
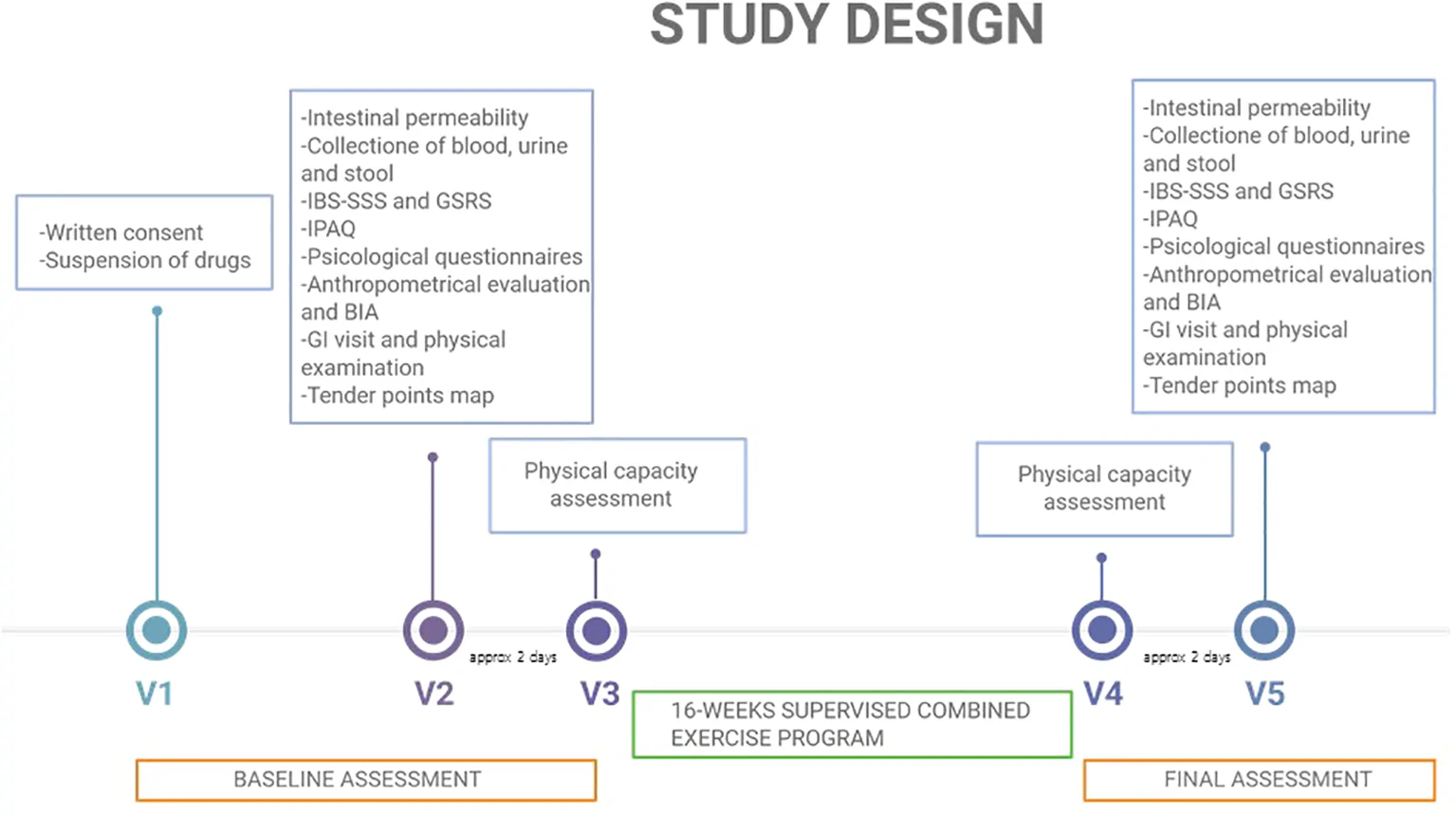
Overview of the study assessment protocol. A timeline of clinical, functional, and laboratory evaluations was administered to all participants upon enrollment. IPAQ, International Physical Activity Questionnaire; IBS-SSS, Irritable Bowel Syndrome Severity Scoring System; BIA, Bioelectrical Impedance Analysis; GI, Gastrointestinal.

This prospective, single-center, interventional pre- and post-study was conducted at the National Institute of Gastroenterology IRCCS “Saverio de Bellis” in Castellana Grotte (Bari), Italy. The study protocol (**Figure 1**) complied with the Declaration of Helsinki and Good Clinical Practice guidelines, received approval from the Institutional Ethics Committee of IRCCS Ospedale Oncologico di Bari (Approval No. 117), and was registered with ClinicalTrials.gov (NCT06166563). All participants provided written informed consent before enrollment.

At V1 (day -14), patients received verbal and written information about the study, signed the informed consent form, and completed a clinical history form; this visit also served to wash out any medication taken for their condition before baseline assessments. At V2, patients underwent blood, urine, and stool collection for laboratory assessments required by the study, and completed questionnaires on GI symptoms, FM-related symptoms, psychological status, quality of life (QoL), and physical activity levels; anthropometric measurements and bioelectrical impedance analysis (BIA) were also performed. At V3, approximately two days after V2, patients obtained medical clearance for non-competitive physical activity, underwent baseline physical capacity field tests, and were instructed by staff specializing in physical activity in the supervised training protocol described in Section 2.4. At V4, approximately two days before V5 and at the end of the 16-week training program, the same field tests were repeated by the same staff, at the same location and time. At V5, patients again completed the GI symptom, FM, psychological, and QoL questionnaires, and blood, urine, stool, anthropometric, and BIA measurements were repeated as at V2.

### 2.2 Participants

Adult patients aged 18–65 years were consecutively recruited from the outpatient units at IRCCS “S. de Bellis”. FM was diagnosed according to the 2010/2011 ACR criteria, as updated in 2016 (Wolfe et al., 2016); IBS was diagnosed according to the Rome IV criteria after systematic exclusion of organic gastrointestinal disease (Drossman, 2016). Exclusion criteria included: autoimmune disease, inflammatory bowel disease, severe psychiatric, neurological, cardiovascular, or hepatic disorders; pregnancy or breastfeeding; use of antibiotics, probiotics, prebiotics, or GI-targeted medications within 6 weeks of enrollment; or inability to participate in supervised physical training. FM and FM+IBS patients were required to have discontinued all pharmacological treatment for fibromyalgia (analgesics, antidepressants, pregabalin, duloxetine, or any centrally active agent) for a minimum of two weeks before enrollment and to remain medication-free throughout the study period.

**Figure 2:**
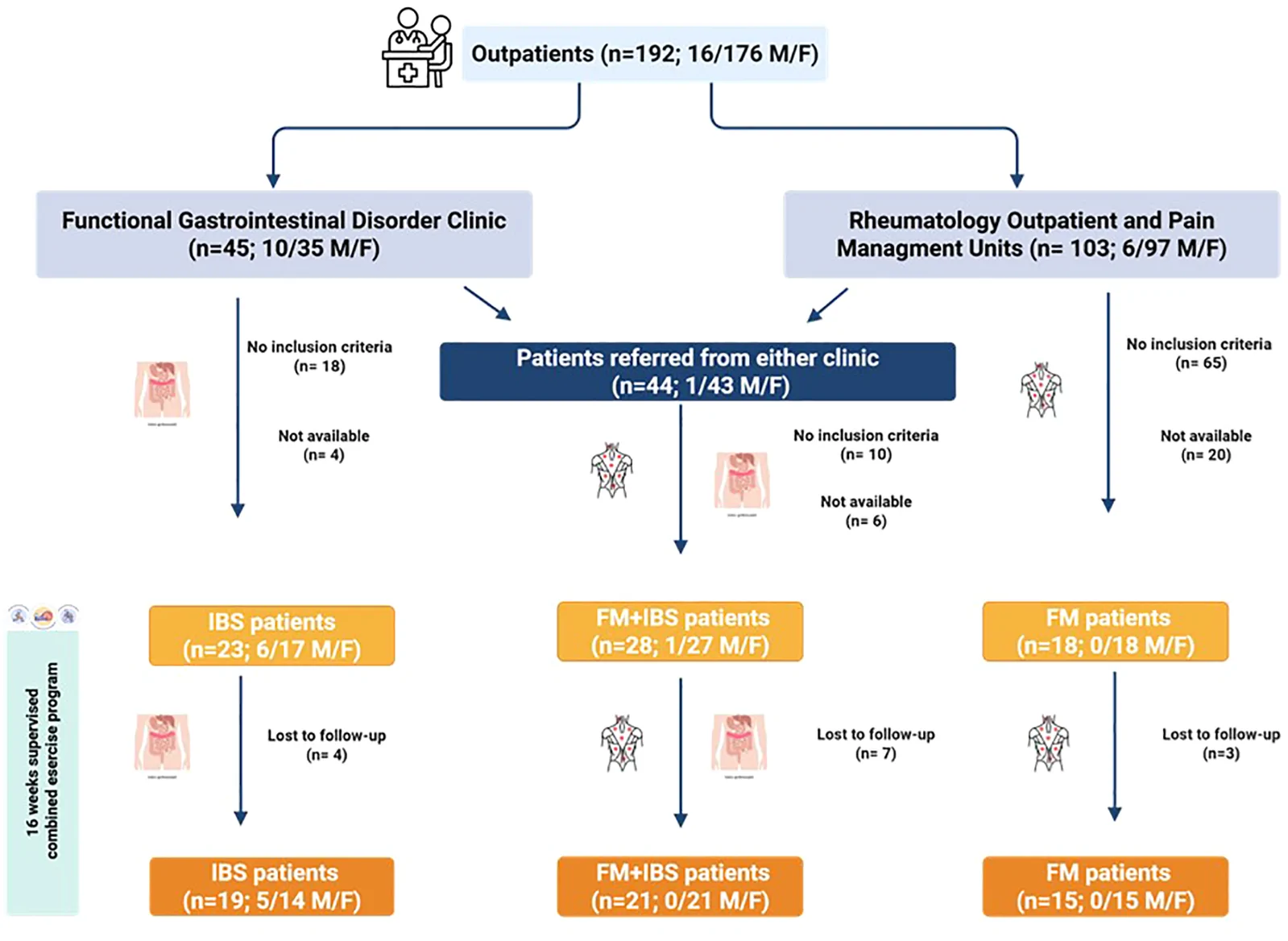
Participant flow through the study. The diagram details the number of individuals screened, eligible, enrolled, and included in the final analysis across the three diagnostic groups: fibromyalgia (FM), irritable bowel syndrome (IBS), and comorbid FM and IBS (FM + IBS). Reasons for loss to follow-up (n= 14) were: 2 relocated, 2 underwent unrelated surgery, 1 domestic accident, 6 change in work commitments, 3 self-reported fatigue with participation.

Of the 192 outpatients initially screened across the Institute’s referral sources (Functional Gastrointestinal Disorder Clinic, n=45; Rheumatology Outpatient and Pain Management Unit, n=103), 123 were excluded prior to enrollment: 93 did not meet the diagnostic inclusion criteria for IBS, FM, or FM+IBS (18, 65, and 10, respectively, according to referral pathway), and 30 were unavailable or unwilling to participate (4, 20, and 6, respectively). The remaining 69 patients were enrolled and allocated to the three diagnostic groups: IBS (n=23), FM (n=18), and FM+IBS (n=28), the latter identified among patients referred from the clinics. During the 16-week supervised exercise program, 14 participants were lost to follow-up (IBS, n=4; FM, n=3; FM+IBS, n=7), yielding a final analytic sample of 55 participants (IBS, n=19; FM, n=15; FM+IBS, n=21). The complete participant flow, including reasons for non-enrollment and attrition by diagnostic group, is detailed in **Figure 2**.

### 2.3 Physical capacity assessment

Patient-reported physical activity (PA) levels were measured using the International Physical Activity Questionnaire - Short Form (IPAQ-SF), a widely validated tool that assesses PA frequency, duration, and intensity over the past week (Lee et al., 2011). During the same week, objective PA levels were assessed using the ActiGraph GT9X accelerometer.

Physical capacity (PC) was assessed objectively using the GPCS composite score. The GPCS combines three validated and reproducible motor tests assessing cardiorespiratory fitness, muscular strength, and flexibility: the 2-kilometer walking test (Laukkanen et al., 1992), the handgrip strength test (Pearn and Bullock, 1979), and the sit-and-reach test (Hoeger and Hopkins, 1992). Each of the three component tests (2-km walking test, handgrip strength test, sit-and-reach test) is scored on a 1–5 scale according pre-established reference tables differentiated by sex and age; the sum of the three scores generated a composite index with a theoretical range from 3 (lowest physical fitness) to 15 (highest fitness)-

Field tests were administered to participants at baseline (V3) and upon completion of the 16-week exercise intervention (V4).

### 2.4 Combined exercise training protocol

The combined exercise training (aerobic, resistance, and flexibility activities) was conducted at an external gym affiliated with the institution, three times per week on non-consecutive days, for 16 weeks. The weekly schedule comprised two session formats: two “combined” sessions, each lasting 60 minutes and sequentially including warm-up, aerobic training, resistance training, flexibility training, and cool-down; and one additional “flexibility” session, preceded by 20 minutes of aerobic exercise on cardio machines and dedicated to joint mobility and flexibility work using both dynamic and static stretching techniques.

Exercise intensity was continuously monitored throughout each session using a heart rate monitor and adjusted according to the Tanaka formula (Tanaka et al., 2001), with perceived exertion assessed using the Borg RPE scale (Wilson and Jones, 1989). Intensity was progressively increased according to each participant’s tolerance and capacity: aerobic training advanced to a moderate range (60-75% of HRmax), and resistance training was progressively loaded up to 6/7 on the Borg scale. Flexibility exercises were always performed within each participant’s pain-free range of motion (ROM), and movements that caused discomfort were avoided.

Exercise volume was reduced when symptoms occurred during or after training sessions, first by decreasing intensity or session duration and, if needed, by reducing training frequency, to maintain a regular training schedule (Kingsley et al., 2010). Professional kinesiologists supervised all training sessions.

### 2.5 Biochemical and functional evaluations

The sugar absorption test (SAT) was performed after an overnight fast; participants ingested lactulose (10 g), mannitol (5 g), and sucrose (40 g) in 100 mL of water, and urine was collected over five hours. Urinary sugars and the L/M ratio were measured by HPLC (L/M >= 0.030 defined as pathological paracellular permeability) (Menzies et al., 1999). Serum and fecal zonulin were measured by ELISA (Immunodiagnostik AG); both assays are subject to cross-reactivity with the complement protein properdin, which inflates absolute values and limits their specificity as zonulin-selective measures; results are interpreted accordingly (Scheffler et al., 2018). Serum I-FABP was quantified by ELISA (Thermo Fisher Scientific). Serum DAO activity was measured by ELISA (ImmunoStep). Urinary indoxyl sulfate (indican) was quantified by a validated colorimetric assay (Indican Assay Kit, ABNOVA Corporation; values >20 mg/L used as a reference threshold for elevated fermentative activity) (Bryan, 1965). Serum LPS, 5-HT, BDNF, IL-6, IL-8, IL-10, and TNF-alpha were measured by ELISA (MyBioSource, Inc.). All assays were performed in duplicate by laboratory personnel blinded to group assignment.

### 2.6 Symptom assessments

FM symptom burden was assessed with the Fibromyalgia Impact Questionnaire-Revised (FIQ-R; range 0-100) (Bennett et al., 2009). IBS symptom severity was assessed with the IBS Severity Scoring System (IBS-SSS; range 0-500) (Francis et al., 1997). The IBS-SSS was administered to all participants, including the FM-only group, to screen for subclinical gastrointestinal symptom burden below the Rome IV threshold for an IBS diagnosis, consistent with our prior observation that FM and IBS symptom domains overlap substantially even in the absence of formal comorbidity (Almansa et al., 2009).

### 2.7 Statistical analysis

The primary outcomes for this proof-of-concept study were the urinary L/M ratio and the IBS-SSS total score, pre-specified as the most direct functional readouts of paracellular permeability and gastrointestinal symptom severity, respectively. All other biomarkers were treated as secondary or exploratory outcomes and should be interpreted as hypothesis-generating. Descriptive statistics are reported as mean ± standard error of the mean (SEM) for continuous variables and as absolute frequencies with percentages for categorical variables. Normality was assessed using the Shapiro-Wilk test. Given the non-normal distributions of most biomarkers and the small group sizes, non-parametric tests were used throughout.

Within-group pre-to-post intervention differences were evaluated using the Wilcoxon matched-pairs signed-rank test. Effect size was calculated as r = Z/sqrt(N), where Z is the standardized test statistic and N is the total number of observations. Values of r = 0.1, 0.3, and 0.5 were interpreted as small, medium, and large effects, respectively.

Between-group comparisons were performed using the Kruskal-Wallis test. When statistically significant, pairwise *post hoc* comparisons were conducted using the Mann-Whitney U test with Bonferroni correction for three pairwise comparisons. Corrected p-values are reported throughout. Between-group comparisons were performed separately for pre-intervention, post-intervention, and delta (post minus pre) values. For the Kruskal-Wallis test, effect size was calculated as eta squared (eta2 = (H - k + 1)/(N - k), where H is the test statistic, k is the number of groups, and N is the total sample size) and is reported for all between-group delta comparisons.

Associations between continuous variables were assessed using Spearman rank correlation (rho). Given the exploratory nature of these analyses, a two-stage approach was adopted. Nominal p-values are reported first, followed by Benjamini-Hochberg false discovery rate (FDR)-adjusted q-values calculated separately within two pre-defined families of tests: (i) the five baseline-versus-delta correlations computed for the barrier-related markers that changed significantly in the FM+IBS group (Section 3.3), and (ii) the five additional exploratory correlations reported in Section 3.8. Correlations with q<0.05 within their respective family are considered to survive correction for multiple comparisons; all others are reported as hypothesis-generating only. The complete correlation matrix, nominal p-values, and FDR q-values are provided in **Supplementary Table 1**.

Statistical significance was set at alpha = 0.05 (two-tailed) for all analyses. No formal *a priori* sample size calculation was performed, given the proof-of-concept nature of the study; findings should therefore be considered exploratory and interpreted with appropriate caution regarding statistical power. *Post hoc* power calculations indicate that, for a paired two-tailed comparison at alpha=0.05, the achieved sample sizes (n=15, 19, and 21 for FM, IBS, and FM+IBS respectively) provide approximately 80% power to detect only large within-group effects (d approximately 0.64-0.78, depending on group size), while power to detect a small-to-medium effect (d=0.3) is only 19-26%. Comparisons that did not reach significance, particularly in smaller subgroups, should therefore be read as inconclusive rather than as evidence of no effect, and are described accordingly throughout the Results. All analyses were performed using SigmaStat 11.0 (Systat Software, San Jose, CA, USA) and GraphPad Prism 9.0 (GraphPad Software, San Diego, CA, USA).

## 3 Results

### 3.1 Participant characteristics

Of 69 patients enrolled, 55 completed all pre- and post-intervention assessments: FM (n=15), FM+IBS (n=21), and IBS (n=19) (**Figure 2**). In the FM and FM+IBS groups, all participants were female; in the IBS group, 5 were male (26.3%). Baseline demographic and anthropometric characteristics (age, BMI) were comparable across groups, whereas IBS-SSS differed significantly, as expected given the diagnostic classification of the cohort (**Table 1**).

**Table 1:** Baseline demographic, anthropometric, and disease-specific characteristics of study participants.

Variable	FM (n=15)	FM+IBS (n=21)	IBS (n=19)	p
Sex (M/F)	0/15	0/21	5/14	–
Age (years)	49.0 ± 2.6	49.4 ± 2.1	53.6 ± 2.1	0.304
BMI (kg/m²)	27.1 ± 2.4	24.2 ± 1.2	26.7 ± 1.2	0.265
GPCS	6.90 ± 0.54	7.02 ± 0.50	8.00 ± 0.52	0.380
IBS-SSS	118.9 ± 23.0	287.3 ± 17.9	219.2 ± 20.6	<0.001*
FIQ-R	61.5 ± 7.6	72.0 ± 7.3	N/A	0.492
Fecal pH	6.64 ± 0.15	6.55 ± 0.10	6.60 ± 0.11	0.721
CRP (mg/L)	0.32 ± 0.11	0.22 ± 0.07	0.29 ± 0.06	0.096
IPAQ low, n (%)	5 (33.3%)	8 (38.1%)	7 (36.8%)	–
IPAQ moderate, n (%)	3 (20.0%)	7 (33.3%)	4 (21.1%)	–
IPAQ high, n (%)	7 (46.7%)	6 (28.6%)	8 (42.1%)	–

Data are presented as mean ± SEM unless otherwise indicated. *p<0.05, Kruskal-Wallis test. BMI, body mass index; GPCS, Global Physical Capacity Score; IBS-SSS, IBS Severity Scoring System; FIQ-R, Fibromyalgia Impact Questionnaire Revised; CRP, C-reactive protein; IPAQ, International Physical Activity Questionnaire. FM patients were medication-free for at least two weeks before enrollment and throughout the study period.

**Figure 3:**
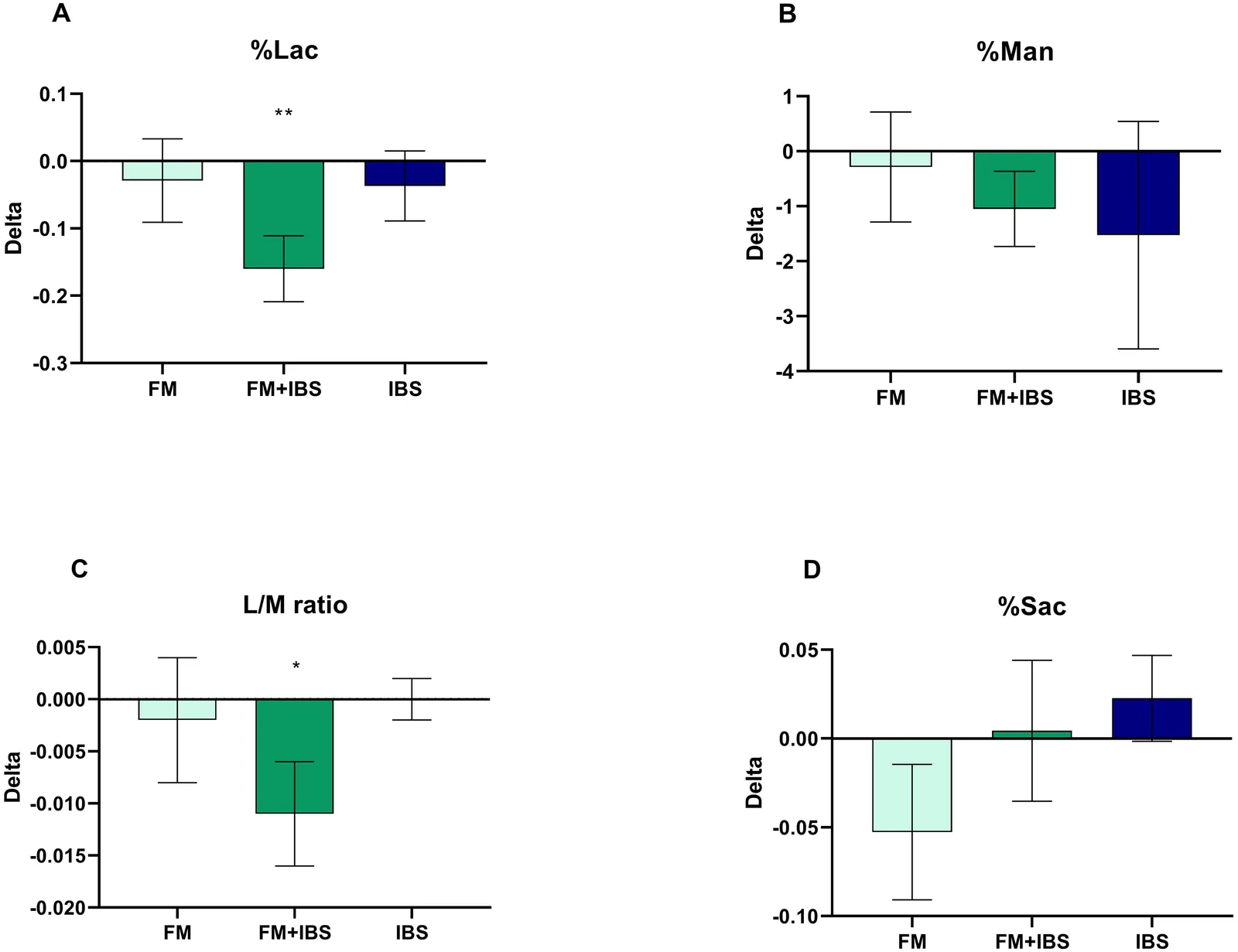
Delta values (post minus pre) for urinary sugar absorption test parameters across the three diagnostic groups (FM, n=15; FM+IBS, n=21; IBS, n=19). Parameters shown: % lactulose excretion, % mannitol excretion, % sucrose excretion, and the lactulose/mannitol (L/M) ratio. Bars represent mean ± SEM. The dashed horizontal line indicates zero. Within-group significance (Wilcoxon matched-pairs signed-rank test): *p<0.05; **p<0.01. Between-group comparisons of delta values using the Kruskal-Wallis test: all p > 0.25. FM, fibromyalgia; IBS, irritable bowel syndrome; L/M, lactulose/mannitol ratio.

**Figure 4:**
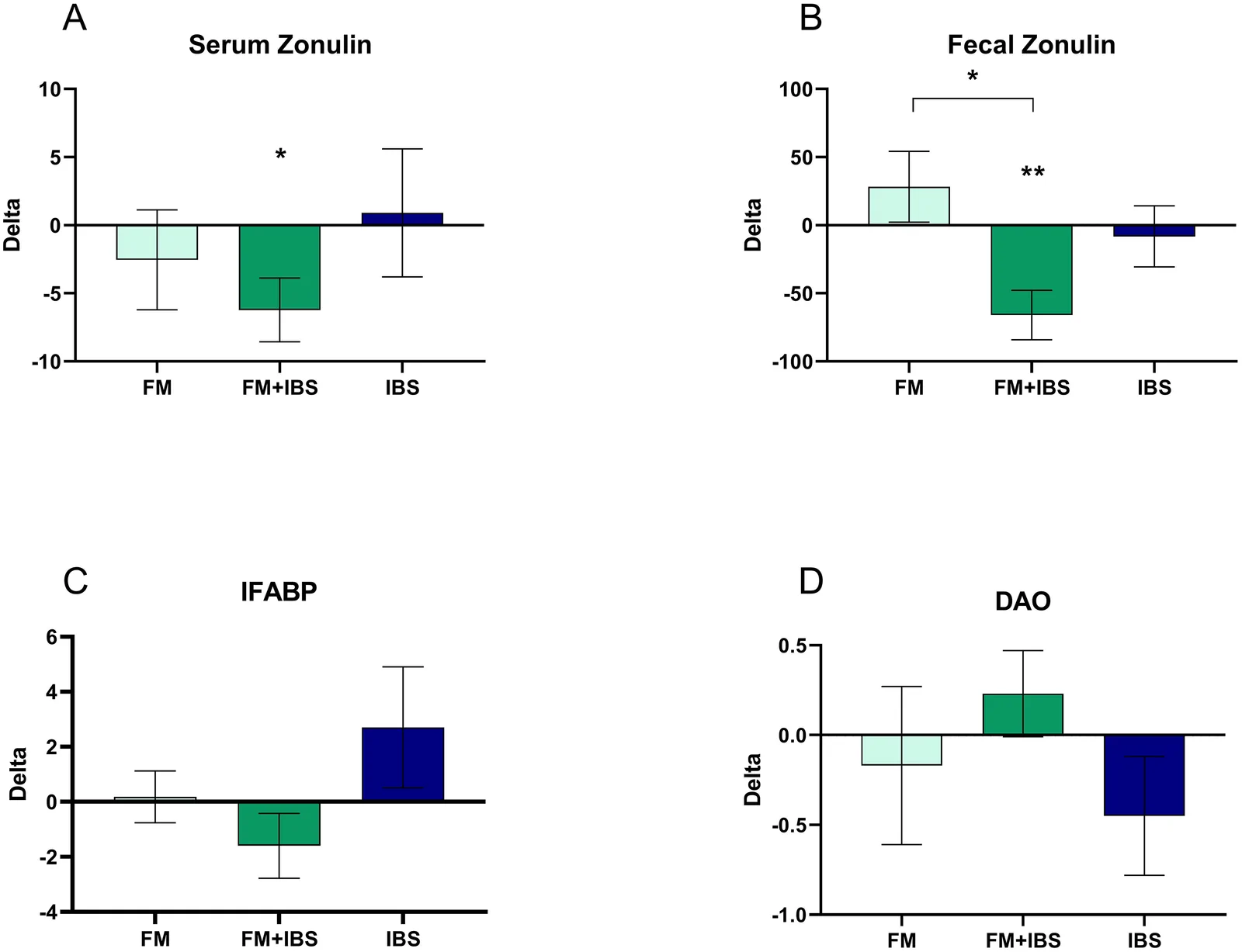
Delta values (post minus pre) for serum zonulin, fecal zonulin, intestinal fatty acid-binding protein (I-FABP), and diamine oxidase (DAO) across the three diagnostic groups (FM, n=15; FM+IBS, n=21; IBS, n=19). Bars represent mean ± SEM. The dashed horizontal line indicates zero. Within-group significance (Wilcoxon matched-pairs signed-rank test): *p<0.05; **p<0.01. Between-group comparisons of delta values by Kruskal-Wallis test: serum zonulin H = 1.98, p=0.372; fecal zonulin H = 8.92, p=0.012; I-FABP H = 2.43, p=0.297; DAO H = 2.46, p=0.292. Bonferroni-corrected *post hoc* comparisons for fecal zonulin delta: FM vs. FM+IBS p=0.016; FM vs. IBS p=1.000; FM+IBS vs. IBS p=0.119. FM, fibromyalgia; IBS, irritable bowel syndrome; I-FABP, intestinal fatty acid-binding protein; DAO, diamine oxidase.

**Figure 5:**
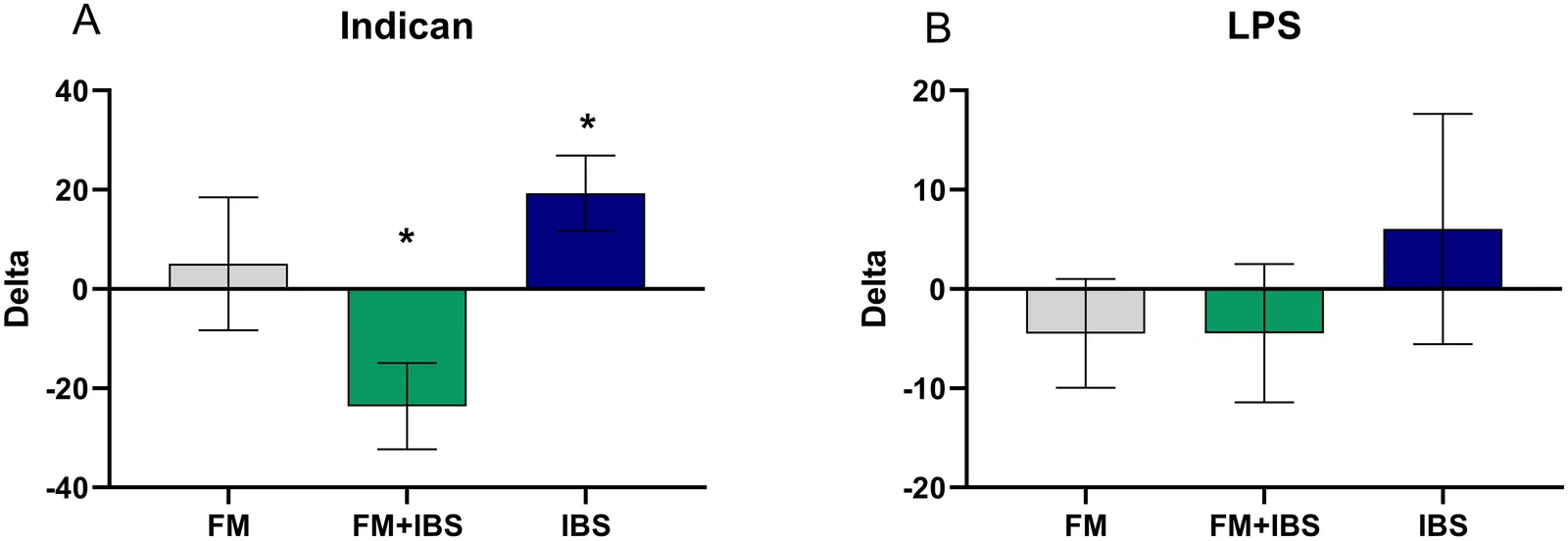
Delta values (post minus pre) for **(A) **urinary indoxyl sulfate (indican) and **(B)** serum lipopolysaccharide (LPS) across the three diagnostic groups (FM, n=15; FM+IBS, n=21; IBS, n=19). Bars represent mean ± SEM. The dashed horizontal line indicates zero. Within-group significance (Wilcoxon matched-pairs signed-rank test): *p<0.05. Between-group comparisons of delta values by the Kruskal-Wallis test: indoxyl sulfate, H = 8.87, p=0.012; LPS, H = 0.03, p=0.984. Bonferroni-corrected *post hoc* comparisons for indoxyl sulfate delta: FM vs. FM+IBS p=0.503; FM vs. IBS p=0.288; FM+IBS vs. IBS p=0.015. FM, fibromyalgia; IBS, irritable bowel syndrome; LPS, lipopolysaccharide.

**Figure 6:**
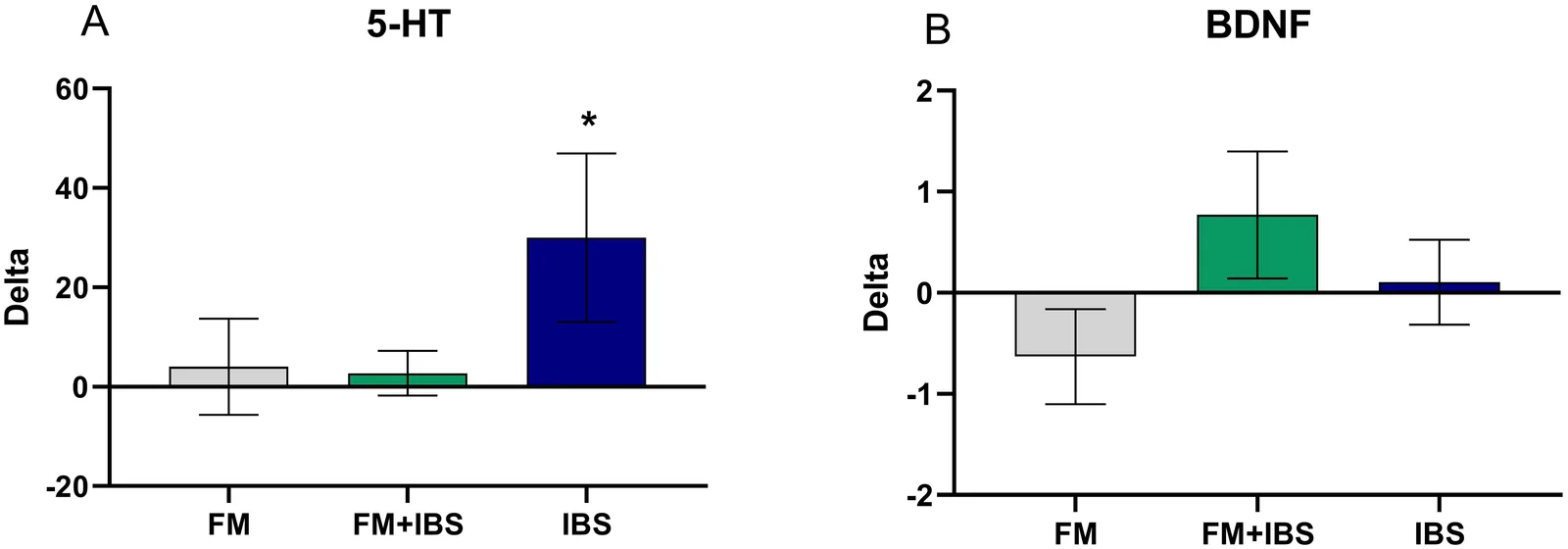
Delta values (post minus pre) for **(A)** serum serotonin (5-HT) and **(B)** brain-derived neurotrophic factor (BDNF) across the three diagnostic groups (FM, n=15; FM+IBS, n=21; IBS, n=19). Bars represent mean ± SEM. The dashed horizontal line indicates zero. Within-group significance (Wilcoxon matched-pairs signed-rank test): *p<0.05. Between-group comparisons of delta values by the Kruskal-Wallis test: 5-HT, H = 4.81, p=0.090; BDNF, H = 3.57, p=0.168. Bonferroni-corrected *post hoc* comparisons: all p>0.18 for 5-HT delta; all p>0.22 for BDNF delta. FM, fibromyalgia; IBS, irritable bowel syndrome; 5-HT, serotonin; BDNF, brain-derived neurotrophic factor.

**Figure 7:**
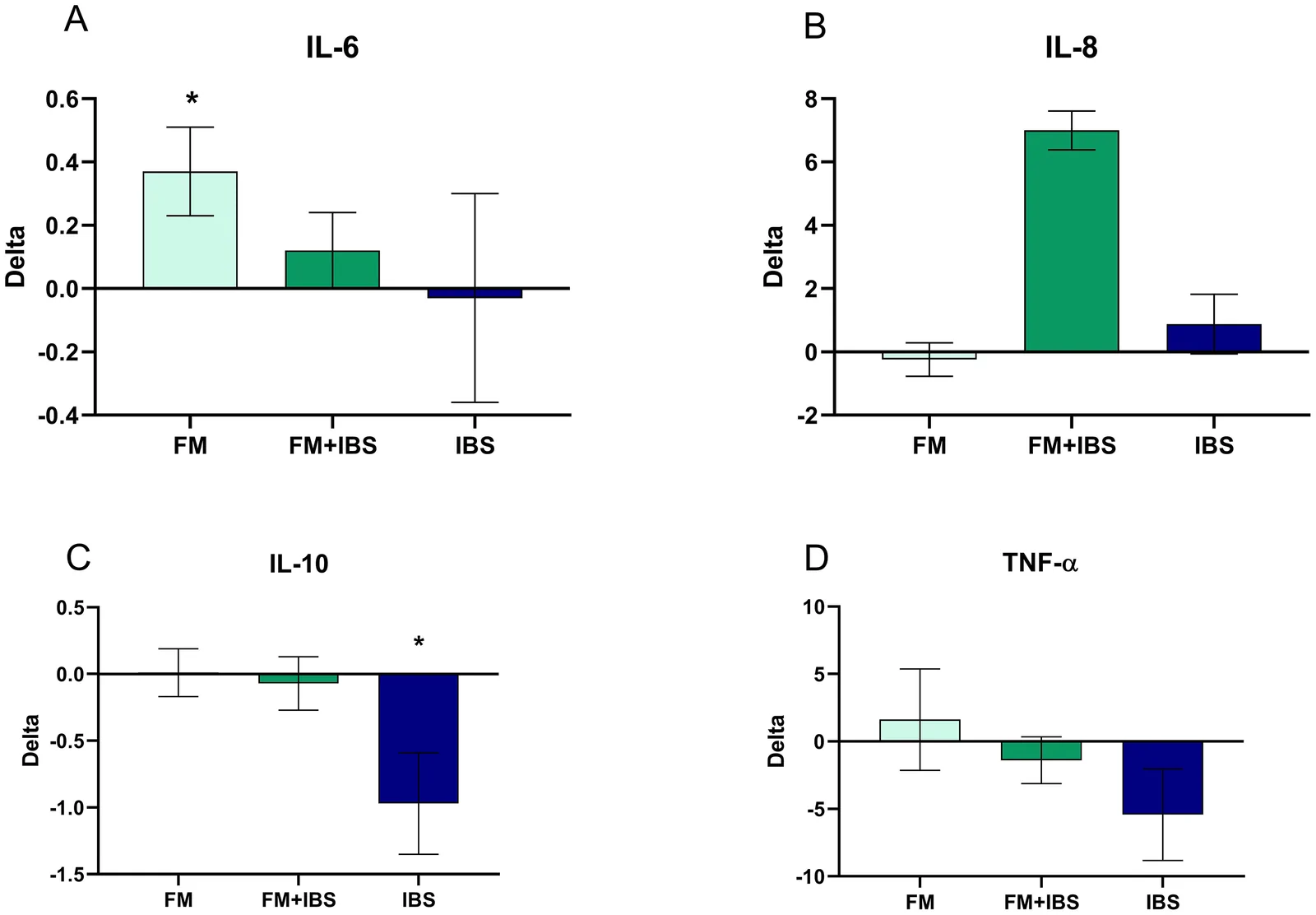
Delta values (post minus pre) for serum interleukin-6 **(A)** (IL-6), interleukin-8 **(B)** (IL-8), interleukin-10 **(C)** (IL-10), and tumor necrosis factor-alpha **(D)** (TNF-alpha) across the three diagnostic groups (FM, n=15; FM+IBS, n=21; IBS, n=19). Bars represent mean ± SEM. The dashed horizontal line indicates zero. Within-group significance (Wilcoxon matched-pairs signed-rank test): *p<0.05. Between-group comparisons of delta values by Kruskal-Wallis test: IL-6 H = 2.71, p=0.258; IL-8 H = 0.70, p=0.705; IL-10 H = 5.65, p=0.059; TNF-alpha H = 1.91, p=0.384. Bonferroni-corrected *post hoc* comparisons for IL-10 delta: FM vs. FM+IBS p=1.000; FM vs. IBS p=0.079; FM+IBS vs. IBS p=0.185. FM, fibromyalgia; IBS, irritable bowel syndrome; IL, interleukin; TNF-alpha, tumor necrosis factor-alpha.

Baseline intergroup differences in barrier-related biomarkers have been reported in detail in a prior publication from our group (Russo et al., 2025). Briefly, FM+IBS patients exhibited significantly greater paracellular permeability and higher zonulin-related protein concentrations than patients with either condition alone. These baseline differences are taken into account throughout the interpretation of post-intervention changes. Pre- and post-intervention absolute values and within-group p-values for all biomarkers are reported in **Table 2**. **Figures 3**–**7** display delta values (post minus pre ± SEM) for each parameter category, with within-group and between-group significance indicated as described below.

**Table 2:** Pre- and post-intervention biomarker values and delta (post-minus-pre) across the three diagnostic groups.

	FM (n=15)			FM+IBS (n=21)			IBS (n=19)			
Biomarker	Pre	Post	p	Pre	Post	p	Pre	Post	p	KW delta p
% Lactulose	0.30 ± 0.03	0.27 ± 0.04	0.363	0.52 ± 0.06	0.36 ± 0.06	0.001**	0.33 ± 0.04	0.29 ± 0.04	0.260	0.250
% Mannitol	13.20 ± 0.98	12.92 ± 0.88	0.524	14.02 ± 0.76	12.97 ± 0.59	0.135	15.19 ± 1.94	13.66 ± 1.16	0.829	0.518
% Sucrose	0.21 ± 0.03	0.16 ± 0.03	0.220	0.32 ± 0.07	0.31 ± 0.07	0.268	0.17 ± 0.03	0.15 ± 0.02	0.813	0.356
L/M Ratio	0.025 ± 0.003	0.023 ± 0.004	0.378	0.038 ± 0.005	0.027 ± 0.004	0.026*	0.023 ± 0.002	0.023 ± 0.002	0.977	0.252
Serum Zonulin (ng/mL)	49.65 ± 3.53	47.11 ± 3.50	0.454	46.98 ± 3.11	40.76 ± 2.24	0.016*	47.66 ± 2.33	48.57 ± 4.66	0.891	0.372
Fecal Zonulin (ng/mL)	165.9 ± 21.8	194.2 ± 28.6	0.330	252.6 ± 17.8	186.7 ± 15.4	0.007**	173.2 ± 18.1	165.0 ± 14.9	0.768	0.012*
I-FABP (pg/mL)	2.91 ± 0.69	3.09 ± 0.52	0.454	6.15 ± 0.99	4.54 ± 1.36	0.218	4.53 ± 1.60	7.22 ± 2.44	0.948	0.297
DAO (U/mL)	8.67 ± 0.79	8.49 ± 0.83	0.594	7.97 ± 0.29	8.21 ± 0.33	0.279	7.92 ± 0.19	7.46 ± 0.26	0.286	0.292
Indoxyl sulfate (mg/L)	52.4 ± 10.0	57.5 ± 10.2	0.804	76.6 ± 9.1	53.0 ± 7.6	0.046*	43.8 ± 3.0	63.1 ± 8.4	0.014*	0.012*
LPS (EU/mL)	108.6 ± 20.6	104.1 ± 21.1	0.804	149.8 ± 23.9	145.4 ± 20.9	0.633	122.5 ± 24.7	128.5 ± 21.4	0.953	0.984
Serotonin (ng/mL)	148.2 ± 10.5	152.2 ± 15.5	0.847	141.3 ± 9.1	144.0 ± 9.8	0.683	150.8 ± 10.3	180.8 ± 22.9	0.028*	0.090
BDNF (ng/mL)	20.00 ± 1.11	19.37 ± 0.89	0.201	21.14 ± 0.8	21.91 ± 0.69	0.151	19.50 ± 0.92	19.61 ± 0.81	0.742	0.168
IL-6 (pg/mL)	0.41 ± 0.07	0.79 ± 0.14	0.013*	0.70 ± 0.13	0.82 ± 0.18	0.387	0.78 ± 0.28	0.75 ± 0.16	0.737	0.258
IL-8 (pg/mL)	5.28 ± 0.87	5.04 ± 0.92	0.345	6.89 ± 1.09	7.59 ± 1.11	0.305	5.82 ± 0.88	6.70 ± 0.93	0.409	0.705
IL-10 (pg/mL)	2.07 ± 0.67	2.08 ± 0.57	0.900	1.43 ± 0.27	1.36 ± 0.22	0.781	2.58 ± 0.67	1.61 ± 0.38	0.025*	0.059
TNF-alpha (pg/mL)	11.85 ± 5.26	13.47 ± 5.56	0.804	14.50 ± 5.61	13.11 ± 5.20	0.794	17.95 ± 5.6	12.53 ± 4.90	0.170	0.384
GPCS	6.90 ± 0.54	8.10 ± 0.57	0.047*	7.02 ± 0.50	8.26 ± 0.42	0.075	8.00 ± 0.52	9.32 ± 0.47	0.039*	0.932
IBS-SSS	118.9 ± 23.0	110.9 ± 18.5	0.955	287.3 ± 17.9	256.6 ± 25.5	0.424	219.2 ± 20	133.3 ± 18.8	<0.001**	0.022*

Data are mean ± SEM. Within-group comparisons by the Wilcoxon matched-pairs signed-rank test. *p<0.05; ** p<0.01. “KW delta p” = Kruskal-Wallis p-value for between-group comparison of delta (post minus pre) values. KW effect size (eta2) = (H − k + 1)/(N − k), where k = 3 groups, N = 55. Significant KW delta comparisons (p<0.05): Fecal Zonulin (H = 8.92, p=0.012, eta2 = 0.133), FM vs. FM+IBS p=0.016; Indoxyl sulfate (H = 8.87, p=0.012, eta2 = 0.132), FM+IBS vs. IBS p=0.015; IBS-SSS (H = 7.63, p=0.022, eta2 = 0.108), FM vs. IBS p=0.023 (Bonferroni-corrected *post hoc* p-values).

### 3.2 Physical capacity and symptom outcomes

GPCS improved significantly in the FM group (6.90 ± 0.54 vs. 8.10 ± 0.57; p=0.047; r=0.51; mean delta: +1.20 ± 0.51) and in the IBS group (8.00 ± 0.52 vs. 9.32 ± 0.47; p=0.039; r=0.47; mean delta: +1.32 ± 0.53), whereas the FM+IBS group showed only a non-significant trend toward improvement (7.02 ± 0.50 vs. 8.26 ± 0.42; p=0.075; mean delta: +1.24 ± 0.60). The absolute magnitude of GPCS change was numerically similar across all three groups (FM: +1.20, FM+IBS: +1.24, IBS: +1.32 points), and between-group comparisons showed no significant differences in delta values (KW H = 0.14; p=0.932; eta2 = 0.00).

The IBS-SSS total score improved significantly in the IBS group (219.2 ± 20.6 vs. 133.3 ± 18.8; p<0.001; r=0.78; mean delta: -85.8 ± 16.0 points), exceeding the minimally important 50-point difference. In the FM+IBS group, the IBS-SSS showed a non-significant decline (287.3 ± 17.9 vs. 256.6 ± 25.5; mean delta: -30.8 ± 29.6 points; p=0.424). In the FM group, the score remained unchanged (118.9 ± 23.0 vs. 110.9 ± 18.5; mean delta: -8.0 ± 21.9 points; p=0.955).

Between-group comparisons confirmed significant differences in IBS-SSS at baseline (KW H = 20.06; p<0.001) and post-intervention (KW H = 19.03; p<0.001). Delta values differed significantly across groups (KW H = 7.63; p=0.022; eta2 = 0.108); *post hoc* comparisons showed that the reduction in IBS was significantly greater than in FM (p=0.023), whereas FM+IBS vs. IBS (p=0.144) and FM vs. FM+IBS (p=1.000) did not survive correction. Results are summarized in **Table 2**.

### 3.3 Paracellular permeability: sugar absorption test

In the FM group (n=15), no significant pre-to-post changes were observed in any permeability parameter (all p>0.22). The mean delta for the L/M ratio was -0.002 ± 0.006 and for %lactulose, -0.029 ± 0.060%.

In the FM+IBS group (n=21), the exercise program was associated with significant reductions in urinary %lactulose (0.515 ± 0.064 vs. 0.355 ± 0.062; p=0.001; r=0.70; mean delta: -0.160% ± 0.048%) and in the L/M ratio (0.038 ± 0.005 vs. 0.027 ± 0.004; p=0.026; r=0.48; mean delta: -0.011 ± 0.005). Post-intervention median L/M values (0.018 [IQR 0.013-0.039]) fell below the 0.030 threshold for pathological paracellular permeability. Given that FM+IBS patients had the highest baseline L/M values in this cohort (Russo et al., 2025), Spearman correlations between baseline values and delta values were computed for the five barrier-related markers that changed significantly in FM+IBS. Strong negative correlations were observed for serum zonulin (rho=-0.703, p<0.001), fecal zonulin (rho=-0.638, p=0.002), and urinary indoxyl sulfate (rho=-0.666, p=0.001), while the correlations for L/M ratio (rho=-0.402, p=0.070) and %lactulose (rho=-0.293, p=0.198) did not reach significance. After Benjamini-Hochberg FDR correction within this family of five tests, serum zonulin (q=0.002), fecal zonulin (q=0.003), and indoxyl sulfate (q=0.002) remained significant, while L/M ratio (q=0.088) and %lactulose (q=0.198) did not survive correction (**Supplementary Table 1**). Neither %mannitol nor %sucrose changed significantly in any group (all p>0.13).

In the IBS group (n=19), no significant pre-to-post changes were observed for any permeability parameter (all p>0.25). Mean deltas were negligible: L/M ratio 0.000 ± 0.002; %lactulose -0.037% ± 0.050%.

Between-group comparisons by Kruskal-Wallis test revealed significant differences in %lactulose (H = 9.41; p=0.009) and L/M ratio (H = 7.30; p=0.026) at baseline. *Post hoc* comparisons for %lactulose showed FM+IBS to be significantly higher than both FM (p=0.028) and IBS (p=0.030), while FM and IBS did not differ (p=1.000). Post-intervention, these differences were no longer significant for either %lactulose (H = 0.25; p=0.885) or L/M ratio (H = 0.25; p=0.883). No significant between-group differences were observed for delta values of any permeability marker (all KW p>0.25; eta2 = 0.00 for all delta comparisons). Results are reported in **Figure 3**.

### 3.4 Intestinal barrier biomarkers: zonulin, I-FABP, and DAO

Serum and fecal zonulin were quantified using the same commercial ELISA kit, which cross-reacts with properdin rather than selectively detecting the pre-haptoglobin 2 zonulin precursor (Scheffler et al., 2018). Accordingly, the two measures are not treated as independent biological replicates in this study; their results are reported below and interpreted jointly as a single, methodologically constrained line of evidence, distinct from the L/M ratio and indoxyl sulfate findings reported in Sections 3.3 and 3.5.

In the FM group, no significant pre-to-post changes were observed for serum zonulin (p=0.454; mean delta: -2.54 ± 3.55 ng/mL), fecal zonulin (p=0.330; mean delta: +28.3 ± 25.1 ng/mL), I-FABP (p=0.454; mean delta: +0.18 ± 0.91 pg/mL), or DAO (p=0.594; mean delta: -0.17 ± 0.42 U/mL).

In the FM+IBS group, serum zonulin decreased significantly (47.0 ± 3.1 vs. 40.8 ± 2.2 ng/mL; p=0.016; r=0.53; mean delta: -6.22 ± 2.28 ng/mL) and fecal zonulin showed a significant reduction (252.6 ± 17.8 vs. 186.7 ± 15.4 ng/mL; p=0.007; r=0.59; mean delta: -65.9 ± 17.8 ng/mL). I-FABP and DAO remained unchanged (p=0.218 and p=0.279, respectively).

In the IBS group, no significant pre-to-post differences were observed for any barrier biomarker (all p > 0.28). Mean deltas: serum zonulin +0.91 ± 4.57 ng/mL; fecal zonulin -8.2 ± 21.8 ng/mL; I-FABP +2.70 ± 2.08 pg/mL; DAO -0.45 ± 0.32 U/mL.

Between-group comparisons showed no significant baseline differences in serum zonulin (KW H = 0.22; p=0.898) or DAO (KW H = 0.21; p=0.902), while fecal zonulin (KW H = 11.68; p=0.003) and I-FABP (KW H = 7.70; p=0.021) differed significantly at baseline. *Post hoc* comparisons for fecal zonulin: FM+IBS was significantly higher than both FM (p=0.014) and IBS (p=0.011), while FM and IBS did not differ (p=1.000). Post-intervention, between-group differences were no longer significant for fecal zonulin (KW H = 0.52; p=0.771), serum zonulin (KW H = 2.84; p=0.242), or I-FABP (KW H = 0.03; p=0.985). Delta values showed a significant overall between-group difference for fecal zonulin (KW H = 8.92; p=0.012; eta2 = 0.133); Bonferroni-corrected *post hoc* comparisons showed the reduction in FM+IBS was significantly greater than in FM (p=0.016), while FM+IBS vs. IBS (p=0.119) and FM vs. IBS (p=1.000) were non-significant. No significant between-group differences in delta values were observed for serum zonulin (KW H = 1.98; p=0.372), I-FABP (KW H = 2.43; p=0.297), or DAO (KW H = 2.46; p=0.292). Results are reported in **Table 2** and **Figure 4**.

### 3.5 Urinary indoxyl sulfate (Indican) and serum LPS

Urinary indoxyl sulfate decreased significantly in the FM+IBS group (76.6 ± 9.1 vs. 53.0 ± 7.6 mg/L; p=0.046; r=0.44; mean delta: -23.6 ± 8.5 mg/L) and increased significantly in the IBS group (43.8 ± 3.0 vs. 63.1 ± 8.4 mg/L; p=0.014; r=0.56; mean delta: +19.3 ± 7.4 mg/L). No significant change was observed in the FM group (p=0.804; mean delta: +5.1 ± 13.0 mg/L). Baseline values differed between FM+IBS (76.6 mg/L, above the 20 mg/L reference threshold) and IBS (43.8 mg/L), and the two groups moved in opposite directions post-intervention.

Between-group comparisons revealed significant baseline differences in urinary indoxyl sulfate (KW H = 7.00; p=0.030). *Post hoc* comparisons showed that FM+IBS was significantly higher than IBS (p=0.030). Post-intervention, between-group differences were no longer significant (KW H = 0.89; p=0.640). Delta values differed significantly across groups (KW H = 8.87; p=0.012; eta2 = 0.132); Bonferroni-corrected *post hoc* comparisons showed a significant difference between FM+IBS and IBS (p=0.015), whereas FM vs. FM+IBS (p=0.503) and FM vs. IBS (p=0.288) were non-significant.

Serum LPS levels remained unchanged across all three groups (FM: p=0.804, FM+IBS: p=0.633, IBS: p=0.953). There were no significant between-group differences at baseline (KW H = 1.33; p=0.514), post-intervention (KW H = 1.68; p=0.432), or for delta values (KW H = 0.03; p=0.984). Results are shown in **Table 2** and **Figure 5**.

### 3.6 Serum 5-HT and BDNF

Serum 5-HT increased significantly only in the IBS group (150.8 ± 10.3 vs. 180.8 ± 22.9 ng/mL; p=0.028; r=0.51; mean delta: +30.0 ± 16.5 ng/mL). No significant change was observed in the FM group (p=0.847; mean delta: +4.0 ± 9.3 ng/mL) or FM+IBS group (p=0.683; mean delta: +2.7 ± 4.4 ng/mL). Between-group comparisons showed no significant differences at baseline (KW H = 0.27; p=0.872), post-intervention (KW H = 1.84; p=0.399), or for delta values (KW H = 4.81; p=0.090; eta2 = 0.05). The IBS group was the only subgroup that included male participants (5 males, 26.3%), whereas both the FM and FM+IBS groups were entirely female.

Because of this sex imbalance, a sensitivity analysis was conducted restricting the IBS group to its 14 female participants. Both principal within-group findings for this subgroup were confirmed: serum 5-HT increased significantly in females only (144.2 ± 12.6 vs. 181.5 ± 30.4 ng/mL; p=0.033; r=0.57; mean delta: +37.4 ± 22.4 ng/mL), and the IBS-SSS reduction remained significant, with a numerically larger effect than in the full mixed-sex group (233.1 ± 22.4 vs. 154.3 ± 21.6; p=0.004; r=0.78; mean delta: -78.9 ± 18.0 points; **Supplementary Table 2**).

BDNF remained stable within each group (FM: p=0.201; FM+IBS: p=0.151; IBS: p=0.742). A nominally significant between-group difference emerged for post-intervention BDNF values (KW H = 7.12; p=0.029); however, Bonferroni-corrected pairwise comparisons did not confirm any specific pair (FM vs. FM+IBS p=0.091; FM+IBS vs. IBS p=0.062; FM vs. IBS p=1.000), and no significant between-group difference was found for delta values (KW H = 3.57; p=0.168; eta2 = 0.030). Results are reported in **Table 2** and **Figure 6**.

### 3.7 Inflammatory markers

IL-6 increased significantly in the FM group (0.41 ± 0.07 vs. 0.79 ± 0.14 pg/mL; p=0.013; r=0.64; mean delta: +0.37 ± 0.14 pg/mL). No significant change in IL-6 was observed in FM+IBS (p=0.387; mean delta: +0.12 ± 0.11 pg/mL) or IBS (p=0.737; mean delta: -0.03 ± 0.32 pg/mL). Between-group comparisons showed no significant differences in IL-6 at baseline, post-intervention, or for delta values (all KW p>0.25).

IL-8 did not change significantly within any group (all p>0.30), and no between-group differences were found at any time point or for delta values (all KW p>0.34).

IL-10 decreased significantly in the IBS group (2.58 ± 0.67 vs. 1.61 ± 0.38 pg/mL; p=0.025; r=0.51; mean delta: -0.97 ± 0.37 pg/mL). This within-group change was not accompanied by any significant between-group difference in delta values (KW H = 5.65; p=0.059; eta2 = 0.06), and the Bonferroni-corrected pairwise comparison between FM and IBS delta values did not survive correction (p=0.079).

TNF-alpha showed no significant pre-to-post changes in any group (all p>0.17) and no significant between-group differences at any time point or for delta values (all KW p>0.38). Results are reported in **Table 2** and **Figure 7**.

### 3.8 Exploratory correlation analyses

All Spearman correlation analyses were conducted exploratorily. Nominal and Benjamini-Hochberg FDR-corrected q-values, computed within the two pre-specified families described in Methods 2.7, are reported below and in full in **Supplementary Table 1**.

A positive association was observed between baseline LPS levels and baseline IBS-SSS total score across the full sample (rho=0.283; p=0.036; q=0.036). In the IBS group, post-intervention GPCS correlated inversely with post-intervention I-FABP (rho=-0.525, p=0.021; q=0.032) and IL-8 (rho=-0.686, p=0.001; q=0.006). IBS-SSS correlated positively with serum DAO at baseline (rho=0.522, p=0.022; q=0.032) and post-intervention (rho=0.509, p=0.026; q=0.032). All five correlations remained significant after Benjamini-Hochberg FDR correction within their pre-specified family (**Supplementary Table 1**).

## 4 Discussion

This study asked a question largely overlooked in the exercise-intervention literature: whether the biological effects of supervised combined training are uniform across functional somatic syndromes, or whether FM, IBS, and their comorbidity each follow a distinct neuroimmune trajectory. Prior studies have established that exercise reduces symptom burden in both FM (Casanova-Rodríguez et al., 2025) and IBS (Johannesson et al., 2011; Nunan et al., 2022; Bianco et al., 2023) when these conditions are examined separately, but none have tracked phenotype-specific changes across a concurrent panel spanning intestinal barrier integrity, serotonergic signaling, neurotrophic activity, and systemic inflammation in the same cohort. Sixteen weeks of training yielded qualitatively distinct biological patterns in each group: FM+IBS patients showed reductions in five barrier-related markers; IBS-only patients exhibited enhanced serotonergic signaling alongside substantial symptomatic improvement; FM-only patients showed myokine-related inflammatory changes and meaningful gains in physical capacity. These patterns are internally consistent with the divergent neuroimmune profiles that characterize each phenotype at baseline and, if confirmed in controlled trials, carry direct implications for individualized exercise prescription in this population.

The most prominent finding is the change in paracellular permeability and barrier-related markers in patients with FM+IBS. Before interpreting this finding, two methodological constraints must be stated upfront. First, serum and fecal zonulin were both measured with the same commercial ELISA kit, which cross-reacts with properdin rather than selectively detecting pre-haptoglobin 2, the true zonulin precursor (Scheffler et al., 2018). Their parallel decline is therefore not independent biological corroboration from two separate compartments, but a single observation obtained twice with a shared assay artifact, and we treat it as such rather than as two converging lines of evidence.

More reliable indicators of permeability change are the urinary L/M ratio, a direct functional measure of paracellular permeability that is not subject to ELISA artifacts, and urinary indoxyl sulfate, which reflects luminal microbial metabolic activity via a distinct mechanism. Both changed significantly in the same direction in FM+IBS patients, and post-intervention L/M values fell below the 0.030 pathological threshold. This is the strongest argument against a pure regression-to-the-mean explanation for the FM+IBS findings: if the observed changes reflected only statistical regression from an extreme baseline, we would not expect two mechanistically unrelated assays to move concordantly, nor would we expect this convergence to respect diagnostic phenotype boundaries so cleanly, given that IBS patients with normal baseline L/M values showed no drift in either direction. We nonetheless cannot rule out regression to the mean as a partial contributor, and the strong negative baseline-delta correlations described below are compatible with either a genuine dose-response relationship or a regression artifact; the present design cannot fully distinguish between the two, and this remains an explicit limitation rather than a resolved question.

None of the candidate upstream mechanisms (HPA-axis reactivity, splanchnic perfusion, or tight junction protein expression) was measured in this trial, and this proof-of-concept study was not designed to adjudicate between them. We note only that exercise-induced modulation of each has precedent in preclinical and early human work (Dokladny et al., 2016; Feng et al., 2021); testing which, if any, applies here requires direct measurement in a future controlled trial and is not pursued further in this Discussion.

The strong negative correlations between baseline values and post-intervention deltas for serum zonulin, fecal zonulin, and indoxyl sulfate (rho=-0.703, -0.638, and -0.666 respectively) all survive Benjamini-Hochberg FDR correction within their test family (q=0.002, 0.003, and 0.002; **Supplementary Table 1**), indicating these are unlikely to be chance findings given the number of correlations tested. However, as noted above, this pattern is equally consistent with a genuine dose-response relationship between baseline dysfunction and treatment response and with regression to the mean, and the current design does not allow us to adjudicate between these explanations.

In patients with IBS only, the primary change was serotonergic. Serum 5-HT rose significantly, and IBS-SSS improved by a mean of 85.8 points, well above the 50-point threshold that defines a clinically meaningful response. A plausible mechanistic link is exercise-induced 5-HT release from enterochromaffin cells, with downstream activation of 5-HT4-mediated propulsive motility, as supported by both experimental and early clinical evidence (Chaouloff, 1989; Gershon and Tack, 2007; Mawe and Hoffman, 2013). IBS patients had normal L/M ratios at baseline, making regression to the mean an implausible explanation for the 5-HT and symptom changes, though this does not substitute for a non-exercising control group.

A related caveat concerns sex composition. The IBS subgroup was the only one that included 5 male participants, which represents an uncontrolled source of variance given the well-established sex differences in serotonergic signaling in IBS, including estrogen-mediated modulation of SERT expression and platelet 5-HT uptake (Franke et al., 2010; van Kessel et al., 2021). A sensitivity analysis restricted to the 14 female IBS patients confirmed both the significant 5-HT increase and the IBS-SSS improvement, with effect sizes numerically similar to or larger than those in the full mixed-sex group. This substantially reduces, though does not entirely eliminate, the concern that the serotonergic and symptomatic findings are an artifact of sex composition; generalizability to a mixed-sex IBS population presenting in other settings should still be considered provisional.

The differing behavior of urinary indoxyl sulfate across groups warrants careful attention. FM+IBS patients, who entered the study with markedly elevated baseline values well above the reference threshold, showed a significant decline, whereas IBS patients, starting from a lower but still abnormal baseline, showed the opposite trend. Rather than diverging further, the two groups converged post-intervention, each approaching the other from its respective starting point. The opposite-direction changes are consistent with, but do not establish, exercise-induced shifts in luminal microbial fermentation; we collected no metagenomic or tryptophan metabolomic data in this trial and flag this strictly as a hypothesis for future, purpose-designed study rather than as an explained mechanism.

In FM-only patients, the dominant biological change was an isolated increase in serum IL-6, without parallel changes in other inflammatory markers. This finding is consistent with multiple physiological explanations, and we do not regard the myokine interpretation as the more likely of the two. The transient exercise-induced myokine response described by Pedersen and Febbraio (Pedersen and Febbraio, 2008) refers specifically to an acute IL-6 surge released by contracting muscle that peaks within hours of a single bout and returns to baseline rapidly; it is not, on its own, a model for a resting, steady-state IL-6 difference measured 16 weeks apart. Chronic training adaptation is, if anything, more commonly associated with a reduction in resting IL-6 as part of an anti-inflammatory training effect. An isolated rise in resting IL-6 without any accompanying change in IL-10 or TNF-alpha is therefore at least as consistent with incomplete recovery, cumulative training stress, or disease-related fluctuation intrinsic to FM as it is with a beneficial myokine-mediated adaptation. Distinguishing between these possibilities would require serial IL-6 sampling around individual training sessions, which this design did not include; we present both interpretations here without favoring one, and flag the myokine explanation as speculative rather than established.

Notably, FM patients also showed significant improvement in objective physical capacity, with a medium-to-large effect size, suggesting that functional adaptation was a meaningful and measurable outcome in this subgroup even in the absence of barrier or serotonergic changes. The absence of significant changes in barrier biomarkers is consistent with the lower baseline permeability burden observed in FM-only patients in our prior cross-sectional analysis (Russo et al., 2025).

The improvement in physical capacity deserves attention in its own right. GPCS improved significantly in both the FM and IBS groups, with medium-to-large effect sizes, while the FM+IBS group showed a numerically similar but non-significant gain, likely reflecting the higher symptom burden and the reduced statistical power of this subgroup. Between-group comparisons revealed no significant differences in the magnitude of functional improvement across phenotypes. This means that despite starkly different biological trajectories in every other domain, all three groups improved their objective fitness to roughly the same degree. Exercise worked as a conditioning stimulus regardless of phenotype; what differed was not the functional response but the biological pathway that carried it out. This parallel structure reinforces the argument that phenotype-specific biological profiling captures information that symptom scores and functional assessments alone cannot reveal. In the IBS group, an exploratory correlation suggested that patients who gained the most in physical capacity also showed the most favorable post-intervention biological profile, though the direction of this relationship cannot be established from the current data. All five correlations in this exploratory family, spanning baseline LPS-IBS-SSS, post-intervention GPCS with I-FABP and IL-8, and IBS-SSS with DAO at both time points, survived Benjamini-Hochberg FDR correction within their pre-specified family, though they remain hypothesis-generating and require confirmation in an independent, adequately powered sample.

BDNF remained unchanged across all groups. Given the moderate exercise intensity used throughout the intervention, this null finding is not surprising. Circulating BDNF responses to exercise appear to require either higher aerobic intensities or longer training periods than those employed here (Szuhany et al., 2015), and peripheral BDNF concentrations are a relatively insensitive index of the central neuroplastic adaptations that matter most in pain sensitization. This outcome should not be read as evidence that BDNF is irrelevant to these conditions, only that the current protocol was not designed to test that hypothesis.

This study has several important limitations, which should be weighed together when interpreting the findings above. The pre-post design without a concurrent control group precludes causal inference, and although the convergence of two independent assays argues against regression to the mean as the sole driver of the FM+IBS paracellular permeability findings, it cannot be ruled out as a partial contributor; all associative language in this manuscript is intended to be read strictly as observational. A related constraint applies to the zonulin data: serum and fecal values share a single cross-reactive ELISA assay and are treated throughout as a single non-independent data point rather than as corroborating evidence from separate compartments. The cohort’s sex composition introduces an additional structural confound, as the FM and FM+IBS groups were exclusively female, whereas the IBS group included five men. The female-only sensitivity analysis mitigates this concern without eliminating it, and generalizability to male FM and FM+IBS patients remains untested. No *a priori* sample size calculation was performed, and the achieved power, approximately 80% for large effects but only 19-26% for small-to-medium effects, means that non-significant comparisons, such as the IL-10 between-group delta, are best read as inconclusive rather than as evidence of equivalence across phenotypes. For that comparison specifically, the achieved power of approximately 20-26% to detect a small-to-medium effect (r~0.3) at these subgroup sizes is a better description than the absence of a between-group IL-10 difference. The isolated IL-6 increase observed in the FM group remains open to two equally plausible readings, a myokine-mediated adaptation or a training- and disease-related fluctuation, that this design cannot distinguish between. Finally, all Spearman correlations reported here are exploratory, and only those surviving Benjamini-Hochberg correction within their pre-specified family are used to support any conclusion in this manuscript.

## 5 Conclusions

This proof-of-concept study provides preliminary, observational evidence that biological responses measured alongside exercise participation in functional somatic syndromes differ by phenotype, arguing against indiscriminately pooling patients with FM and IBS in future interventional research. Despite comparable observed gains in objective physical capacity across all three groups, the underlying neuroimmune trajectories diverged in ways consistent with each phenotype’s baseline biology: changes in permeability-related markers in FM+IBS (supported most directly by the L/M ratio and indoxyl sulfate), serotonergic and symptomatic improvement in IBS (confirmed in a female-only sensitivity analysis), and an isolated, mechanistically ambiguous IL-6 change in FM. In the absence of a non-exercising control group, none of these findings can be interpreted causally. These findings support the design of a phenotype-stratified, adequately powered, controlled randomized trial and, as a hypothesis for future testing, suggest that exercise prescriptions for these patients may ultimately need to be calibrated to the individual’s biological profile, not just to symptom burden or functional capacity.

## Data Availability

The datasets presented in this study can be found in online repositories. The names of the repository/repositories and accession number(s) can be found below: 10.6084/m9.figshare.32725677.
