# Supplementary material for: Divergent intestinal barrier and neuroimmune responses to supervised exercise across fibromyalgia, irritable bowel syndrome, and their comorbidity phenotypes: a prospective interventional study
Source: Front Physiol. 2026 Aug 21;17:1912788. doi: 10.3389/fphys.2026.1912788 (PMC13541565; doi:10.3389/fphys.2026.1912788)
Supplement: Supplementary file 1 [file Table1.docx]

**Supplementary Material**

*Manuscript: Divergent Intestinal Barrier and Neuroimmune Responses to Supervised Exercise Across Fibromyalgia, Irritable Bowel Syndrome, and Their Comorbidity Phenotypes: A Prospective Interventional Study (Manuscript ID 1912788, Frontiers in Physiology)*

**Supplementary Table S1. Full correlation matrix with FDR-corrected q-values**

All Spearman correlations reported in the manuscript (Sections 3.3 and 3.8), with nominal p-values and Benjamini-Hochberg FDR-adjusted q-values computed separately within two pre-defined families: Family A (baseline-versus-delta correlations for the five barrier markers that changed significantly in FM+IBS, Section 3.3) and Family B (additional exploratory correlations, Section 3.8). q<0.05 within a family is considered to survive correction.

| **Family** | **Correlation** | **Group / n** | **rho** | **Nominal p** | **FDR q** | **Survives** |
| --- | --- | --- | --- | --- | --- | --- |
| A | Baseline serum zonulin vs. delta serum zonulin | FM+IBS, n=21 | -0.703 | <0.001 | 0.002 | Yes |
| A | Baseline fecal zonulin vs. delta fecal zonulin | FM+IBS, n=21 | -0.638 | 0.002 | 0.003 | Yes |
| A | Baseline indoxyl sulfate vs. delta indoxyl sulfate | FM+IBS, n=21 | -0.666 | 0.001 | 0.002 | Yes |
| A | Baseline L/M ratio vs. delta L/M ratio | FM+IBS, n=21 | -0.402 | 0.070 | 0.088 | No |
| A | Baseline %lactulose vs. delta %lactulose | FM+IBS, n=21 | -0.293 | 0.198 | 0.198 | No |
| B | Baseline LPS vs. baseline IBS-SSS | Full sample, n=55 | 0.283 | 0.036 | 0.036 | Yes |
| B | Post GPCS vs. post I-FABP | IBS, n=19 | -0.525 | 0.021 | 0.032 | Yes |
| B | Post GPCS vs. post IL-8 | IBS, n=19 | -0.686 | 0.001 | 0.006 | Yes |
| B | Baseline DAO vs. baseline IBS-SSS | IBS, n=19 | 0.522 | 0.022 | 0.032 | Yes |
| B | Post DAO vs. post IBS-SSS | IBS, n=19 | 0.509 | 0.026 | 0.032 | Yes |

*BH FDR correction performed using the Benjamini-Hochberg procedure (statsmodels 'fdr_bh' method), applied separately within each family as specified in Methods 2.7.*

**Supplementary Table S2. Sex-stratified sensitivity analysis, IBS group**

Within-group pre-to-post comparisons for serum 5-HT and IBS-SSS in the full IBS group (n=19, including 5 male participants) and in the female-only IBS subgroup (n=14), conducted to assess whether the serotonergic and symptomatic findings reported in Section 3.6 are attributable to the sex composition of the group.

| **Outcome** | **Subgroup** | **n** | **Pre (mean±SEM)** | **Post (mean±SEM)** | **Delta (mean±SEM)** | **p (Wilcoxon)** | **r** |
| --- | --- | --- | --- | --- | --- | --- | --- |
| Serum 5-HT (ng/mL) | Full IBS | 19 | 150.8 ± 10.3 | 180.8 ± 22.9 | +30.0 ± 16.5 | 0.028 | 0.51 |
| Serum 5-HT (ng/mL) | Female-only IBS | 14 | 144.2 ± 12.6 | 181.5 ± 30.4 | +37.4 ± 22.4 | 0.033 | 0.57 |
| IBS-SSS (points) | Full IBS | 19 | 219.2 ± 20.6 | 133.3 ± 18.8 | -85.8 ± 16.0 | <0.001 | 0.78 |
| IBS-SSS (points) | Female-only IBS | 14 | 233.1 ± 22.4 | 154.3 ± 21.6 | -78.9 ± 18.0 | 0.004 | 0.78 |
